# Diagnostic and Therapeutic Challenges in an Older Patient With Concurrent Small-Cell Lung Carcinoma and Primary Duodenal Adenocarcinoma: A Case Report

**DOI:** 10.7759/cureus.83040

**Published:** 2025-04-26

**Authors:** Ryuichi Ohta, Kaoru Tanaka, Masayuki Miyata, Junko Tanizaki, Hidetoshi Hayashi

**Affiliations:** 1 Community Care, Unnan City Hospital, Unnan, JPN; 2 Department of Medical Oncology, Kindai University Faculty of Medicine, Sayama, JPN

**Keywords:** adjuvant, aged, chemotherapy, duodenal neoplasms, elderly, multiple primary neoplasms, positron emission tomography-computed tomography, small-cell lung carcinoma

## Abstract

An 80-year-old woman presented with weight loss, elevated pro-gastrin-releasing peptide levels, and later developed exertional dyspnea. Contrast computed tomography of the chest revealed multiple masses in the left upper lobe of the lung, left hilar and mediastinal lymphadenopathy, and pleural effusion, suggesting pleural dissemination. Additionally, positron emission tomography-computed tomography showed fluorodeoxyglucose uptake in the descending portion of the duodenum and associated wall thickening. Histopathological examination of the bronchoscopic biopsy specimens demonstrated small to intermediate-sized tumor cells with scant cytoplasm, finely granular chromatin, and nuclear molding, supporting the diagnosis of small-cell lung cancer (SCLC). She was diagnosed with extensive-stage SCLC and experienced gastrointestinal bleeding from the suspected duodenal metastasis lesion before the treatment, with severe anemia. She was promptly started on systemic chemotherapy with carboplatin, etoposide, and durvalumab due to clinical urgency and suspicion of duodenal metastasis. Although the pulmonary tumor responded to treatment, gastrointestinal bleeding persisted. The endoscopic biopsy confirmed primary duodenal adenocarcinoma. Surgical resection was not pursued due to the patient’s condition, and chemotherapy for SCLC was continued. This case illustrates the importance of synchronous primary cancers in patients with atypical presentations and highlights the need to balance prompt treatment with thorough diagnostic evaluation. Early treatment of the more aggressive cancer allowed clinical stabilization and outpatient management, demonstrating a pragmatic approach to complex oncologic care in elderly patients.

## Introduction

Small-cell lung cancer (SCLC) is one of the most aggressive forms of lung malignancy, characterized by rapid progression and a high tendency to metastasize both within the lungs and to extrapulmonary sites [1]. More recent data often estimate the proportion closer to 13-15%, as the incidence has slightly declined over the past few decades, likely due to reduced smoking rates and earlier detection of non-small-cell lung cancer [2]. Due to its high sensitivity to chemotherapy and radiotherapy, prompt initiation of treatment is crucial to improve outcomes and manage symptoms effectively [3].

However, the rapid and widespread nature of SCLC can pose significant diagnostic challenges, particularly when evaluating extrapulmonary lesions [4]. Distinguishing between metastatic lesions and a second primary malignancy can be difficult, especially when initial biopsy results are inconclusive or have limited sensitivity [5,6]. In such cases, clinicians often prioritize treatment initiation over obtaining definitive pathological differentiation.

In this report, we present the case of an 80-year-old patient diagnosed with extensive-stage SCLC (ES-SCLC) who developed progressive gastrointestinal bleeding. Given the unclear origin of a duodenal lesion and the urgency of her clinical condition, she was initially treated with intensive chemotherapy with an immune checkpoint inhibitor for SCLC. A subsequent biopsy later confirmed the lesion to be a primary duodenal cancer. This case highlights the diagnostic and therapeutic challenges in managing concurrent malignancies and emphasizes the importance of strategic clinical decision-making when faced with overlapping symptoms and ambiguous pathological findings.

## Case presentation

An 80-year-old woman was referred to our hospital for further evaluation of progressive weight loss and elevated serum pro-gastrin-releasing peptide (ProGRP) levels, raising suspicion for an underlying pulmonary malignancy. However, as there was no infiltration in the lungs, the symptoms were followed by her primary care physicians. Approximately 120 days before the presentation, she began experiencing unintentional weight loss of 3 kg per year and consulted her primary care physician. Laboratory tests revealed elevated ProGRP, prompting a referral to a general hospital. At that time, a computed tomography (CT) scan of the chest showed no evident abnormalities, and she was placed under observation.

However, around nine days before her initial visit to our hospital, she developed exertional dyspnea. In the general hospital, a repeat evaluation of chest X-ray revealed a left-sided pleural effusion, and follow-up CT imaging showed a suspicious mass in the left lung, leading to her referral to our facility. Her medical history included a benign breast mass under surveillance at the general hospital. She did not take any medications. She had a significant smoking history (50 pack-years), drank alcohol socially, lived alone, and had no family history of cancer.

On the initial encounter at our hospital, she was alert and oriented, with a blood pressure of 132/82 mmHg, a heart rate of 83 beats per minute, a respiratory rate of 18 breaths per minute, the body temperature of 36.6°C, and an oxygen saturation of 96% on room air. Physical examination revealed mild pallor of the palpebral conjunctivae and late crackles audible in the left lung field, without cardiac murmurs or other remarkable findings. Laboratory data showed mild anemia with a hemoglobin level of 10.3 g/dL; ProGRP levels remained elevated (Table 1).

**Table 1:** Initial laboratory data of the patient. CEA: carcinoembryonic antigen; CRP: C-reactive protein; CYFRA: cytokeratin 19 fragment; Ig: immunoglobulin; ProGRP: pro-gastrin-releasing peptide

Parameter	Level	Reference
White blood cells	9.41	3.5–9.1 × 10^3^/μL
Neutrophils	71.9	44.0–72.0%
Lymphocytes	21.0	18.0–59.0%
Hemoglobin	12.4	11.3–15.2 g/dL
Mean corpuscular volume	94.7	79.0–100.0 fl
Platelets	45.1	13.0–36.9 × 10^4^/μL
Total protein	6.8	6.5–8.3 g/dL
Albumin	3.8	3.8–5.3 g/dL
Aspartate aminotransferase	23	8–38 IU/L
Alanine aminotransferase	18	4–43 IU/L
Lactate dehydrogenase	344	121–245 U/L
Blood urea nitrogen	19	8–20 mg/dL
Creatinine	0.70	0.40–1.10 mg/dL
Serum Na	143	135–150 mEq/L
Serum K	3.2	3.5–5.3 mEq/L
Serum Cl	97	98–110 mEq/L
CRP	0.126	<0.30 mg/dL
CEA	11.3	0-5.0 ng/mL
CYFRA	6.0	0–3.5 ng/mL
ProGRP	3,603	<50 pg/mL
Urine test	-	-
Leukocyte	Negative	Negative
Protein	Negative	Negative
Blood	Negative	Negative

**Figure 1:**
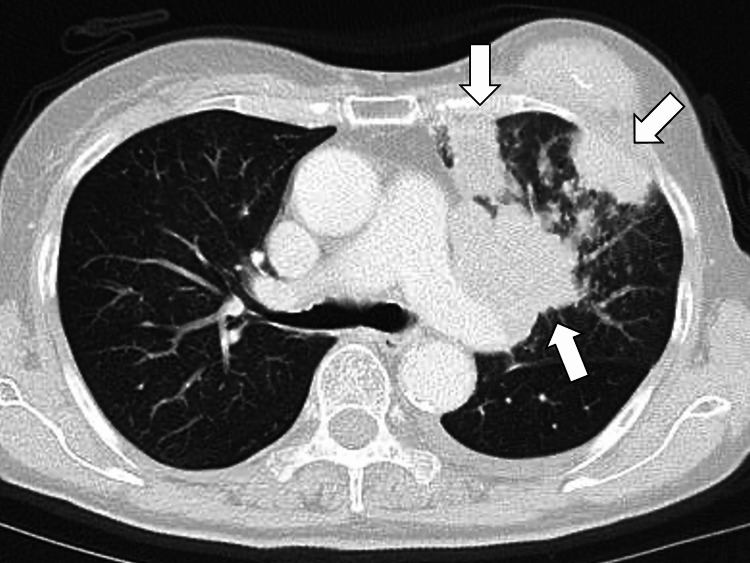
Chest computed tomography revealing multiple masses in the left upper lobe of the lung, associated with left hilar and mediastinal lymphadenopathy (white arrows).

On the same day, chest X-ray, contrast-enhanced CT, and positron emission tomography-computed tomography (PET-CT) were performed. The CT revealed multiple masses in the left upper lobe of the lung, left hilar and mediastinal lymphadenopathy, and pleural effusion, suggesting pleural dissemination (Figure 1).

**Figure 2:**
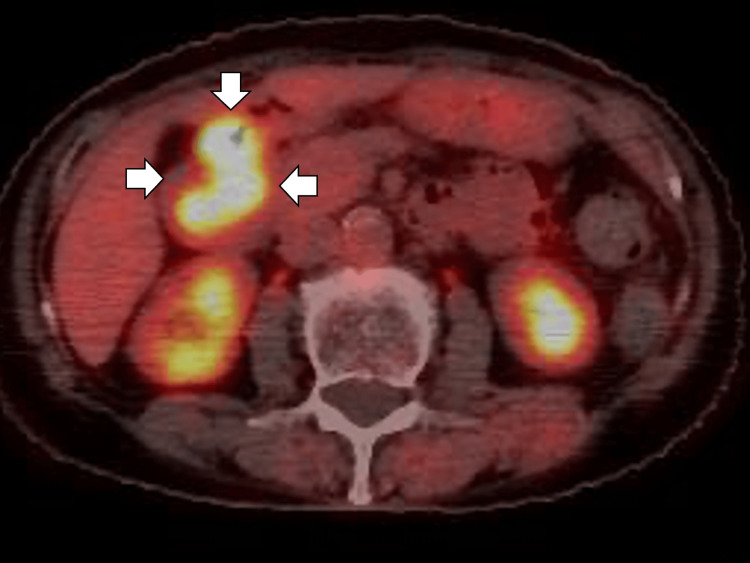
The positron emission tomography-computed tomography showing fluorodeoxyglucose uptake in the descending portion of the duodenum, with a maximum standardized uptake value of 15.8 and associated wall thickening, raising suspicion of a second primary malignancy (white arrows).

Additionally, PET-CT showed fluorodeoxyglucose (FDG) uptake in the descending portion of the duodenum, with a maximum standardized uptake value (SUVmax) of 15.8 and associated wall thickening, raising suspicion of a second primary malignancy (Figure 2).

**Figure 3:**
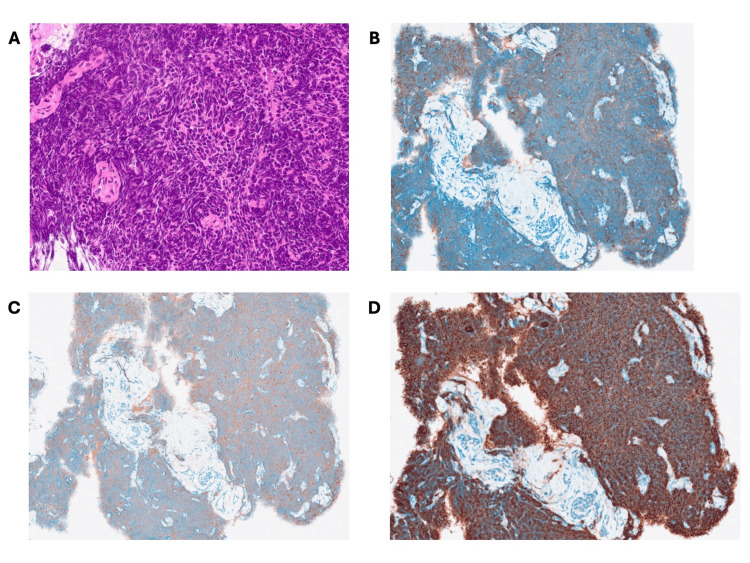
Histopathological examination of the bronchoscopic biopsy specimens demonstrated small to intermediate-sized tumor cells with scant cytoplasm, finely granular chromatin, and nuclear molding in hematoxylin and eosin stain (A). Immunohistochemical staining was positive for neuroendocrine markers such as chromogranin A (B), synaptophysin (C), and CD56 (D), supporting the diagnosis of small-cell lung cancer.

On day six, she underwent bronchoscopy, which was complicated by transient hypoxemia that resolved with conservative management, including intravenous ceftriaxone of 2 g for five days. Histopathological examination of the bronchoscopic biopsy specimens demonstrated small to intermediate-sized tumor cells with scant cytoplasm, finely granular chromatin, and nuclear molding. Immunohistochemical staining was positive for neuroendocrine markers such as synaptophysin, chromogranin A, and CD56, supporting the diagnosis of SCLC (Figure 3).

Multiple enlarged lymph nodes and a small volume of left pleural effusion were noted near the aortic arch. No definitive distant metastases were observed.

On day seven, the case was discussed in a multidisciplinary lung cancer board meeting and based on the findings of pleural effusion, suspected pleural dissemination, and possible carcinomatous lymphangitis, a diagnosis of ES-SCLC was confirmed. Hence, systemic chemotherapy with carboplatin (area under the curve: 5), etoposide (80 mg/m²), and durvalumab (1,500 mg/body) was planned.

**Figure 4:**
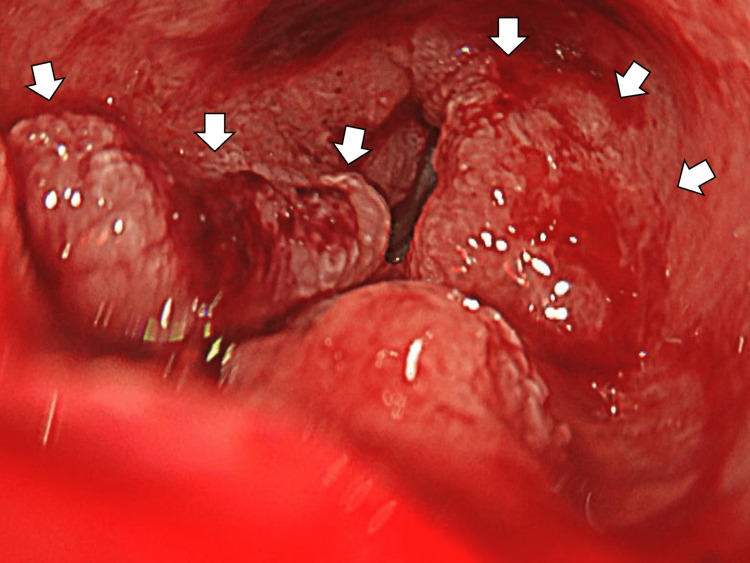
An esophagogastroduodenoscopy revealing a bleeding lesion in the duodenum (white arrows).

On day 18, she was admitted emergently following a syncopal episode. Her hemoglobin had decreased to the 7 g/dL range, raising suspicion of gastrointestinal bleeding. She received a transfusion with four units of red blood cells. On day 19, an esophagogastroduodenoscopy revealed a bleeding lesion in the duodenum (Figure 4).

Biopsy at that time showed no malignant findings and was interpreted as an adenoma. However, given the FDG uptake on PET-CT and clinical scenario, duodenal metastasis of SCLC remained a consideration.

On days 24 to 26, she received the first cycle of systemic chemotherapy consisting of carboplatin (area under the curve: 5), etoposide (80 mg/m²), and durvalumab (1,500 mg). This led to clinical improvement and radiographic regression of the pulmonary lesion.

**Figure 5:**
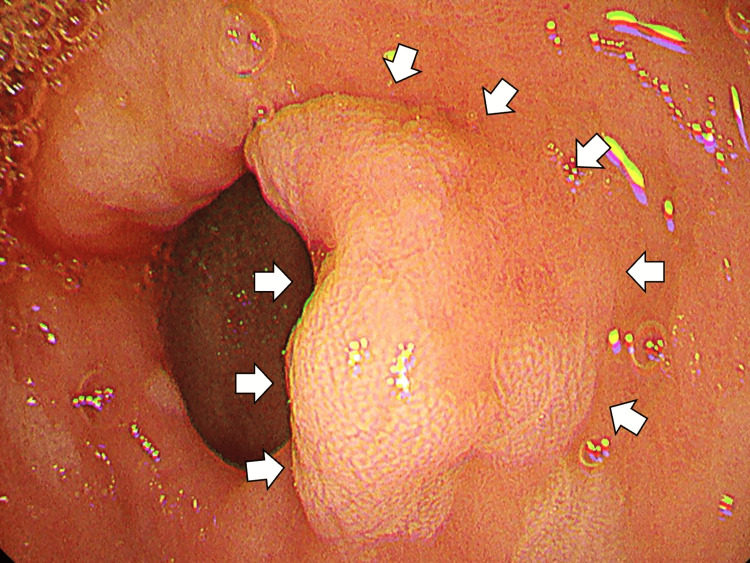
Repeat upper gastrointestinal endoscopy clarifying that the duodenal lesion had not regressed (white arrows).

On day 47, a repeat upper gastrointestinal endoscopy was performed to reassess the duodenal lesion (Figure 5).

**Figure 6:**
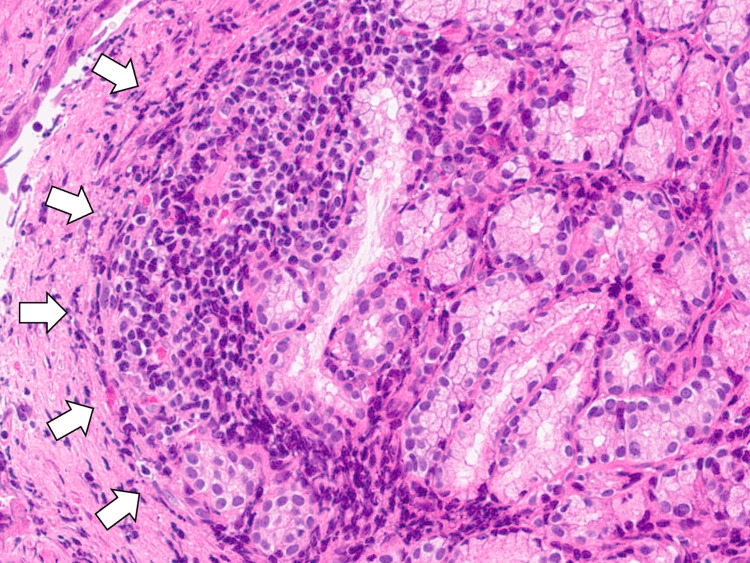
The biopsy of the duodenal lesion confirming the diagnosis of tubular/papillary adenocarcinoma of the duodenum (white arrows), indicating a synchronous second primary malignancy.

The lesion had not regressed, and biopsy confirmed the diagnosis of tubular/papillary adenocarcinoma of the duodenum, indicating a synchronous second primary malignancy (Figure 6).

Surgical consultation was obtained, and while pancreatoduodenectomy was initially considered, it was ultimately deemed too high-risk due to the patient’s advanced age and clinical condition. She completed four cycles of systemic chemotherapy. On day 112, she was evaluated in our outpatient department, remained clinically stable, and continued maintenance therapy with durvalumab.

## Discussion

This case demonstrates the complexity of diagnosing and managing multiple coexisting malignancies in older patients, mainly when clinical urgency necessitates treatment before definitive pathological confirmation. The presence of a hypermetabolic duodenal lesion in a patient with newly diagnosed ES-SCLC raised concern for either metastatic disease or a synchronous primary malignancy, two distinct possibilities with overlapping clinical presentations.

Although SCLC has a well-documented tendency for widespread dissemination, gastrointestinal metastases, especially to the duodenum, are rare in clinical practice despite their higher incidence in autopsy series [7,8]. In our case, the patient’s anemia and gastrointestinal bleeding, combined with the FDG-avid duodenal lesion on PET-CT, initially supported the possibility of metastatic SCLC. However, a non-diagnostic initial biopsy left uncertainty regarding the nature of the lesion.

Given the patient’s rapid clinical decline and high tumor burden in the lung, chemotherapy for ES-SCLC was prioritized. ES-SCLC is typically managed with platinum-based chemotherapy combined with a programmed death-ligand 1 inhibitor, such as atezolizumab or durvalumab, as established by pivotal trials. This combination has demonstrated objective response rates of approximately 60-70%, median progression-free survival of 5.2-5.6 months, and median overall survival of 12-13 months [9]. These outcomes, while improved compared to chemotherapy alone, highlight the aggressive nature of ES-SCLC and the necessity for prompt treatment initiation in symptomatic patients. This pragmatic approach led to symptom relief and radiographic response in the thoracic disease, reinforcing the value of early systemic therapy in ES-SCLC [10]. Notably, the duodenal lesion persisted despite treatment, prompting re-evaluation. A repeat endoscopic biopsy ultimately revealed a primary tubular/papillary adenocarcinoma, confirming the coexistence of a second primary malignancy rather than a metastatic lesion.

This diagnostic course underscores the limitations of initial biopsies and the importance of maintaining diagnostic flexibility. The concept of “anchoring bias,” in which clinicians may overly focus on a single unifying diagnosis, is particularly relevant in oncologic care, where patients are at increased risk for multiple primary cancers [11]. Our findings are consistent with previous reports emphasizing the importance of re-biopsy and reassessment when the clinical course deviates from expected treatment responses [12,13].

In older adults, managing concurrent cancers requires balancing diagnostic thoroughness with timely treatment, especially when one malignancy is immediately life-threatening. Our decision to prioritize ES-SCLC treatment reflected this balance, allowing for symptom control and preservation of quality of life. Moreover, this case highlights the increasing need to individualize care in older patients, considering physiological reserve, goals of care, and risk-benefit assessments of invasive procedures such as surgery for duodenal cancer [14].

Although literature explicitly addressing the co-occurrence of SCLC and primary duodenal adenocarcinoma is limited, broader studies and case reports on synchronous lung and gastrointestinal malignancies offer valuable insights. For example, a case report described successful disease control in a patient with lung adenocarcinoma and synchronous duodenal cancer using a combination of targeted therapy and S-1 chemotherapy [15]. Furthermore, retrospective studies suggest that synchronous malignancies are associated with worse survival outcomes, underlining the importance of comprehensive evaluation and tailored treatment strategies [16,17]. These findings support a multidisciplinary approach integrating oncologic priorities, patient condition, and diagnostic uncertainty into shared decision-making frameworks. Further research and systematic reviews are needed to establish evidence-based management guidelines for complex clinical scenarios.

The concurrent occurrence of SCLC and duodenal adenocarcinoma is exceedingly rare and raises the question of potential genetic predispositions. Although germline or somatic genetic analyses were not performed in this case, it is noteworthy that SCLC is almost universally associated with inactivating mutations in the tumor protein p53 (*TP53*) and retinoblastoma 1 (*RB1*) genes, each present in over 90% of cases [4,18]. Duodenal adenocarcinoma, by contrast, exhibits a distinct mutational profile, including alterations in Kirsten rat sarcoma viral oncogene homolog (*KRAS*) (30-50%), *TP53* (40-60%), adenomatous polyposis coli (*APC*) (20-30%), phosphatidylinositol-4,5-bisphosphate 3-kinase catalytic subunit alpha (*PIK3CA*) (10-20%), and inactivation of SMAD family member 4 (*SMAD4*) (15-30%) [19]. Among these, *TP53* is the only gene commonly mutated in both malignancies. This raises the theoretical possibility of an underlying germline *TP53* mutation, as seen in Li-Fraumeni syndrome, a hereditary cancer predisposition syndrome caused by germline *TP53* mutations [20]. However, the patient had no personal or family history of malignancy, making a hereditary cancer syndrome less likely. Instead, the prolonged history of tobacco use may have contributed to somatic *TP53* mutations, serving as a shared environmental risk factor in the development of both cancers.

Ultimately, clinicians should be cautious not to assume all lesions represent metastases from a known primary cancer. Especially in patients with atypical presentations or persistent lesions after therapy, the possibility of a second primary malignancy should be carefully considered.

## Conclusions

This case highlights the diagnostic and therapeutic challenges in managing synchronous SCLC and primary duodenal adenocarcinoma, particularly in older patients with overlapping clinical symptoms. Initial suspicion of duodenal metastasis delayed the diagnosis of a second primary tumor, emphasizing the need for ongoing reassessment when treatment response is atypical. Early systemic chemotherapy for SCLC stabilized the patient’s condition, allowing further evaluation. Clinicians should remain vigilant for coexisting malignancies and adopt a multidisciplinary, individualized approach to treatment prioritization. This case underscores the importance of balancing diagnostic accuracy with prompt intervention in complex oncologic presentations.
